# Cardiotoxicity of CPX-351 in children and adolescents with relapsed AML: a Children's Oncology Group report

**DOI:** 10.3389/fcvm.2024.1347547

**Published:** 2024-06-14

**Authors:** Kasey J. Leger, Michael J. Absalon, Biniyam G. Demissei, Amanda M. Smith, Robert B. Gerbing, Todd A. Alonzo, Hari K. Narayan, Betsy A. Hirsch, Jessica A. Pollard, Bassem I. Razzouk, Kelly D. Getz, Richard Aplenc, E. Anders Kolb, Bonnie Ky, Todd M. Cooper

**Affiliations:** ^1^ Division of Pediatric Hematology/Oncology, Seattle Children’s Hospital, University of Washington, Seattle, WA, United States; ^2^ Department of Pediatrics, Oregon Health Sciences University, Portland, OR, United States; ^3^ Division of Cardiology, Perelman School of Medicine, University of Pennsylvania, Philadelphia, PA, United States; ^4^ Children’s Oncology Group, Monrovia, CA, United States; ^5^ Department of Population and Public Health Sciences, University of Southern California, Los Angeles, CA, United States; ^6^ Department of Pediatrics, University of California San Diego, Rady Children’s Hospital San Diego, La Jolla, CA, United States; ^7^ Department of Pediatrics, University of Minnesota, Minneapolis, MN, United States; ^8^ Division of Pediatric Oncology, Dana-Farber Cancer Institute, Boston, MA, United States; ^9^ Department of Pediatrics, Harvard Medical School, Boston, MA, United States; ^10^ Department of Pediatrics, Peyton Manning Children’s Hospital at Ascension St. Vincent, Indianapolis, IN, United States; ^11^ Departments of Biostatistics, Epidemiology & Informatics and Pediatrics, Perelman School of Medicine, University of Pennsylvania, Philadelphia, PA, United States; ^12^ Division of Oncology, The Children’s Hospital of Philadelphia, Philadelphia, PA, United States; ^13^ Nemours Center for Cancer and Blood Disorders, A.I. DuPont Hospital for Children, Wilmington, DE, United States

**Keywords:** CPX-351, pediatric acute myeloid leukemia (AML), relapse, cardiotoxicity, liposomal anthracycline, cardiac biomarkers, AAML1421

## Abstract

**Introduction:**

Anthracyclines are effective in treating acute myeloid leukemia (AML) but limited by cardiotoxicity. CPX-351, a liposomal daunorubicin and cytarabine, may provide therapeutic benefit with less cardiotoxicity. Acute changes in left ventricular systolic function and cardiac biomarkers were evaluated after a cycle of CPX-351 in children with relapsed AML treated on the phase 1/2 Children's Oncology Group study, AAML1421.

**Methods:**

Subjects received 135 units/m^2^/dose of CPX-351 on days 1, 3, and 5 as cycle 1. Echocardiograms were performed and centrally quantitated at baseline and at the end of cycle 1 (day 29 +/− 1 week). High sensitivity troponin (hs-cTnT) and N-terminal pro-B-type natriuretic peptide (NT-proBNP) were measured at baseline and serially through the end of cycle 1 (days 5, 8, 15, 22 and 29). Differences between baseline and post-CPX-351 echo/biomarker measures were analyzed using Wilcoxon signed rank tests. Linear regression was used to model post-CPX-351 left ventricular ejection fraction (LVEF) with cTnT/NT-proBNP at each time point, controlling for baseline LVEF. Cancer therapy related cardiac dysfunction (CTRCD) was defined as a decline in LVEF of ≥10%–<50%.

**Results:**

Twenty-five of 38 heavily anthracycline pre-treated (median 348 mg/m^2^ daunorubicin equivalents) subjects enrolled on AAML1421 were included in the cardiac analyses. At baseline, centrally quantitated LVEF was <50% in 8 of 25 subjects (32%) with a median LVEF of 53.8% [48.0, 56.9]. Following CPX-351, LVEF declined significantly (ΔLVEF −3.3% [−7.8, 0]) and 6 of 25 subjects (24%) experienced CTRCD. Amongst all subjects, hs-cTnT was modestly increased at end of cycle 1 compared to baseline [baseline hs-cTnT 7.2 (3, 10.6); ΔcTnT 1.80 (0, 6.1), *p* = 0.03]. NT-proBNP remained stably elevated without significant change. No significant associations were seen between NT-proBNP or cTnT levels and post-CPX-351 LVEF.

**Discussion:**

In this single arm study of anthracycline pre-treated children exposed to CPX-351, baseline abnormalities in cardiovascular function were prevalent. Following CPX-351, LVEF decreased, cTnT increased, and NT-proBNP did not change. Longer follow-up is needed to determine whether these changes result in clinically meaningful long-term decrements in cardiac function. An ongoing randomized trial of CPX-351 compared to standard anthracyclines in anthracycline naïve patients will provide further insight into the cardiac effects of CPX-351 (ClinicalTrials.gov; NCT04293562).

## Graphical Abstract

Baseline and post-CPX-351 abnormalities in cardiac function & biomarkers in children with relapsed pediatric AML.

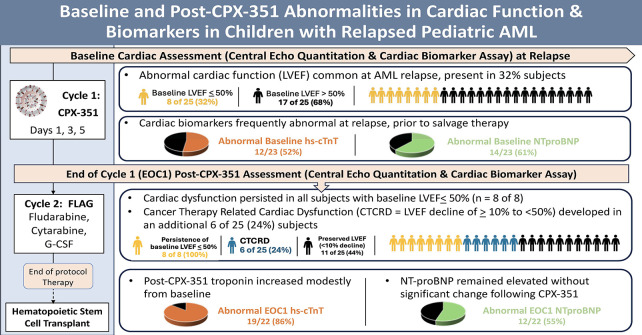

## 1 Introduction

Clinical outcomes of pediatric AML have improved over time with intensification of therapy, but significant risk for leukemia relapse and therapy-related morbidity and mortality remain. High-dose anthracyclines are an integral component of *de novo* AML therapy and contribute to a substantial burden of cardiotoxicity in this patient population. Early cardiotoxicity manifesting as left ventricular systolic dysfunction is common, occurring in 12% of patients at the end of therapy and nearly 20% within 3.5 years after completion of therapy (1). Late cardiomyopathy is also a well-recognized complication of high dose anthracycline therapy, occurring in up to 27% of childhood AML survivors who are 15 years from their initial diagnosis (2). Anthracyclines are an effective component of relapse therapy; however their use is limited by the high risk for deterioration in cardiac function, particularly in a heavily pretreated population with high prior cumulative anthracycline exposure. Therefore, effective, less cardiotoxic salvage therapies for relapsed AML, a population at increased risk for cardiomyopathy and heart failure, are critical.

Liposomal encapsulation of anthracyclines is one strategy employed to reduce cardiotoxicity while also enhancing anti-leukemic efficacy through prolonged drug half-life and exposure. Liposomal delivery systems reduce drug distribution through the tight capillary junctions of the heart and into the cardiac tissue, thereby limiting drug-induced cardiac injury (3). Adult studies of liposomal daunorubicin or doxorubicin have demonstrated a reduction in clinical and subclinical cardiac dysfunction compared to standard anthracycline formulations (4). CPX-351 is a liposomal formulation of daunorubicin and cytarabine maintained at a 1:5 molar ratio that demonstrates prolonged exposures compared with free drug. This agent has demonstrated safety and superior efficacy in adult patients with newly diagnosed secondary AML. While early studies in adults indicate reduced cardiotoxicity of CPX-351 compared to standard daunorubicin (5, 6), there are limited pediatric data regarding the cardiotoxic profile of CPX-351.

AAML1421 was a Children's Oncology Group (COG) phase I/II study of CPX-351 that sought to determine the phase 2 dose and the response rate of CPX-351 in children with relapsed AML. The efficacy and safety of this regimen has been previously reported (7). We now describe the baseline cardiac status and early changes in centrally quantitated imaging and biomarker-based measures of cardiovascular function, injury and stress after a single cycle of CPX-351 in children with relapsed AML, a secondary aim of the AAML1421.

## 2 Methods

### 2.1 Clinical trial design

AAML1421 (ClinicalTrials.gov; NCT04293562) enrolled children between 1 and ≤21 years with relapsed or refractory AML in a dose-finding phase (*n* = 6) followed by children in first relapse in the efficacy phase (*n* = 32). Eligibility was restricted to subjects with previous receipt of <450 mg/m^2^ prior daunorubicin equivalents, calculated using the historic conversion multiplier of 3 daunorubicin equivalents for mitoxantrone. Additionally, subjects must have demonstrated adequate cardiac function based on local assessment, defined as left ventricular (LV) fractional shortening (LVFS) > 27% and LV ejection fraction (LVEF) > 50%, and corrected QT (QTcB) interval <500 milliseconds. Children with CNS3 AML involvement were not eligible due to limited penetration of CPX-351 through the blood brain barrier. Additionally children with acute promyelocytic leukemia, bone marrow failure syndromes, Down syndrome, or Wilson disease were also ineligible.

All subjects received CPX-351 in cycle 1 at a dose of 135 units/m^2^/dose over 90 min on days 1, 3, and 5 during both the dose finding and efficacy phases. Each unit of CPX-351 contains 0.44 mg of daunorubicin, thus the cumulative daunorubicin dose delivered during cycle 1 was 178.2 mg/m^2^. Subjects were recommended to receive cycle 2 consisting of fludarabine, cytarabine, G-CSF (FLAG). Subjects then went off protocol therapy to undergo hematopoietic stem cell transplant (HSCT) or other disease directed therapy at the discretion of the treating physician (Graphical Abstract). A primary objective of the trial was to estimate the response rate after 2 cycles of protocol therapy. Institutional review board approval was obtained at each participating site. All subjects or their guardians were required to provide informed consent prior to participation. Optional consent was provided for participation in the cardiac correlative studies. CPX-351 was supplied by Jazz Pharmaceuticals.

### 2.2 Cardiac studies

Transthoracic echocardiograms were required in all participants at baseline and at end of cycle 1, the CPX-351 containing cycle, approximately 4 weeks from the start of CPX-351. Optional embedded cardiac studies included submission of DICOM echocardiogram studies for central quantitation at baseline and at the end of cycle 1 along with serial plasma collection for cardiac biomarker analysis.

### 2.3 Echocardiogram quantitation

Quantitation of echocardiograms was performed by a single blinded observer at the University of Pennsylvania Center for Quantitative Echocardiography (Philadelphia, PA) using the TomTec Imaging Systems platform. Left ventricular end-diastolic and end-systolic volumes were calculated using the Simpson's method of discs in the 4-chamber and 2-chamber apical views and were utilized to derive LVEF. Cancer therapy–related cardiac dysfunction (CTRCD) was defined as a ≥10% absolute decline in LVEF and to a value <50% (8). In addition, longitudinal and circumferential strain were analyzed using images obtained in the apical four-chamber and two-chamber views, and parasternal short-axis view (SAX) at the mid-papillary level. The LV endocardial border was manually traced at the end-systolic frame of one cardiac cycle. Peak systolic longitudinal strain was automatically quantified across the apical (inferior, septal, anterior, and lateral), mid (inferior, inferoseptal, anterior, and anterolateral) and basal (inferior, inferoseptal, anterior, and anterolateral) LV segments of the apical four-chamber and two-chamber views. Peak circumferential and radial strain were quantified across the mid (inferior, inferoseptal, inferolateral, anterior, anteroseptal, and anterolateral) LV segments of the parasternal SAX view. Reproducibility analyses for the University of Pennsylvania Core Lab have demonstrated intra-observer coefficients of variation were 4.9%, 10.9%, and 9.4% for LVEF, longitudinal strain, and circumferential strain (CS), respectively (8).

### 2.4 Cardiac biomarker analysis

Peripheral blood was collected in a labeled serum separator tube at baseline and on days 5 (6 h after the final dose of CPX-351), 8, 15, 22, and 29 of cycle 1 for analysis of cardiac biomarkers. High sensitivity troponin (hs-cTnT) was measured using the fifth-generation Elecsys Troponin T Gen 5 STAT assay on the Cobas platform (Roche Diagnostics). Abnormal hs-cTnT was defined by published age and sex based 97.5th percentile threshold for children below 18 years and for those 18 and older, using the standard sex-specific adult 99th percentile threshold for the hs-cTnT assay (>14 ng/L for females and >22 ng/L for males) (9). NT-proBNP was measured using the Elecsys ProBNP II STAT Immunoassay on the Cobas platform. Abnormal NT-proBNP was defined using the published 97th percentile for age and sex for those younger than 18 years and the established adult threshold of >125 pg/ml for those 18 years and older (9, 10).

### 2.5 Statistics

Data were frozen for these analyses as of 31 March 2020. All subjects had completed protocol treatment by the analysis cutoff date. The cardiac analyses were restricted to participants with an analyzable LVEF at baseline and end of CPX-351 cycle. Clinical characteristics, including age and weight classification at study entry, prior anthracycline exposure, duration of prior remission, prior HSCT status, and grade 3 or higher infection status during CPX-351 cycle, were summarized using proportions for categorical variables, or medians (ranges) when presented as continuous measurements. Weight categories were defined based on CDC pediatric growth chart data for subjects 1–19 years old or body mass index percentile for subjects 20 years old or higher (underweight, <5th percentile; healthy weight, 5th to <85th percentile; overweight, 85th to <95th percentile; obese, >95th percentile). NT-proBNP measurements were log base 2 transformed due to high dispersion. Troponin measurements below the lower limit of detection (<6 ng/L) were assigned a value of 3 (midpoint between lower limit of detection and zero) to allow their inclusion in the analyses. Differences in cardiovascular measures between baseline and post-CPX-351 were analyzed using Wilcoxon signed rank tests. The significance of observed differences in proportions was tested using Fisher's exact test. Linear regression was used to model the association between post-CPX-351 LVEF with cTnT/NT-proBNP at each post-CPX-351 timepoint separately, adjusting for baseline LVEF and the baseline biomarker being analyzed. These analyses were exploratory and not adjusted for multiple comparisons.

## 3 Results

Thirty-eight subjects enrolled and received cycle 1 of CPX-351. All subjects were in first relapse. Of these, 25 (66%) subjects consented to participate in the cardiac sub-study and provided echocardiographic images with analyzable LVEF at baseline and end of cycle 1. Of the 13 subjects excluded from the cardiac analysis, six failed to provide consent or the site did not submit DICOM echocardiographic images for central analysis. An additional 7 subjects were excluded from cardiac correlatives due to unanalyzable LVEF at baseline or end of cycle 1 (Supplementary Figure S1). Subjects included in the cardiac sub-study were slightly younger and more frequently male and of White race than those who were excluded. Included subjects more commonly experienced grade 3+ sepsis during cycle 1. Otherwise, the two populations were similar (Table 1). Of the 25 subjects included in the cardiac analyses, 11 (44%) were female, 7 (28%) had undergone a prior HSCT and 17 (68%) were within one year of first remission. The median prior anthracycline exposure was 348 mg/m^2^ of daunorubicin equivalents (range 105–444).

**Table 1:** Characteristics of subjects included in and excluded from the cardiac correlative studies on AAML1421.

	Subjects included in cardiac analyses		Subjects excluded from cardiac analyses	
	*N* or median	% or range	*N* or median	% or range
Total	25		13	
Age at study entry in years	12.5	(1.81–21.5)	8.1	(1.93–21.2)
Gender				
Male	14	56%	4	31%
Female	11	44%	9	69%
Race				
Black or African American	1	5%	3	30%
White	19	95%	7	70%
Other or unknown	5	20%	3	23%
Ethnicity				
Hispanic	5	20%	2	15%
Not hispanic or unknown	20	80%	11	85%
Duration of first remission				
<180 days	4	16%	1	8%
180–365 days	13	52%	6	46%
>365 days	8	32%	6	46%
Prior hematopoietic stem cell transplant	7	28%	3	23%
Weight category				
Underweight	1	4%	0	0%
Healthy weight	15	60%	7	54%
Overweight	3	12%	3	23%
Obese	6	24%	3	23%
Cumulative anthracycline exposure at enrollment (mg/m^2^ in daunorubicin equivalents)	348	(105–444)	311	(150–444)
Site reported ejection fraction at study entry				
Measurement available	22	88%	13	100%
Median (range)	62%	(55%–71%)	63%	(54%–76%)
Grade 3+ infection during cycle 1	13	52%	4	31%

### 3.1 Changes in cardiac function, as defined by echocardiography

**Figure 1:**
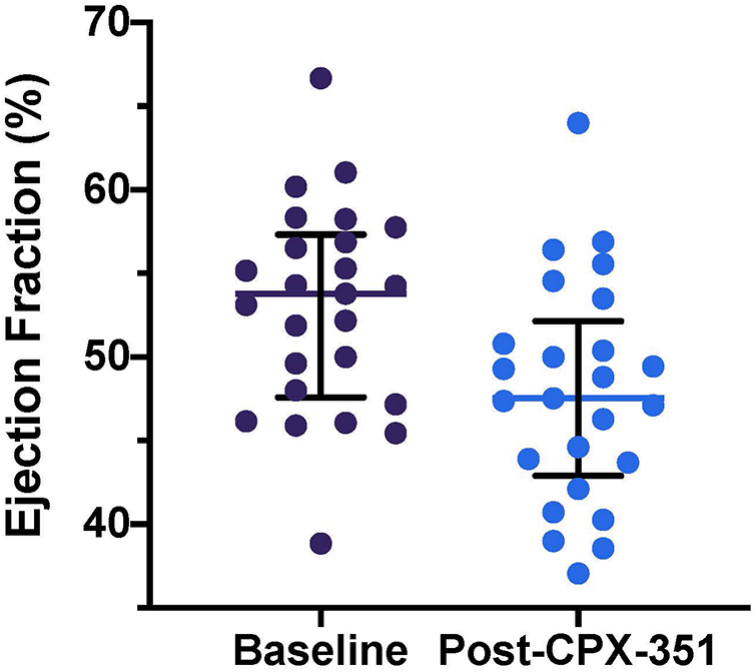
Baseline and post-CPX-351 cycle LVEF measurements. Individual patient LVEF measurements plotted with overlying median and interquartile range for each time point. LVEF declined significantly from baseline at the end of the CPX-351 cycle [ΔLVEF, −3.3% (IQR: −7.8, 0%), *p* < 0.0001].

Despite the inclusion criteria for this study requiring a locally measured baseline LVEF of at least 50%, centrally quantitated LVEF was less than 50% at study entry in 8 of 25 subjects (32%), with abnormal LVEF ranging from 38.9% to 49.6%. On average, across the study population, centrally analyzed LVEF declined from baseline at the end of the CPX-351 cycle {median baseline LVEF, 53.8% [interquartile range (IQR) 48.0, 56.9]; end of CPX-351 cycle, 47.5% [IQR: 43.7, 50.8%], ΔLVEF, −3.3% [IQR: −7.8, 0%], *p* < 0.0001; Figure 1}. Cancer therapy–related cardiac dysfunction (CTRCD), defined as >10% decline to EF < 50%, occurred in 6 of 25 (24%) after CPX-351 (amongst this subset, median baseline LVEF 57.4% and post-CPX-351 LVEF 43%). Subjects with CTRCD were primarily female [5 of 6 (83%) with CTRCD vs. 6 of 19 (32%) without CTCRD, *p* = 0.056] and frequently experienced grade 3+ infection during cycle 1 [5 of 6 (83%) with CTCRD vs. 8 of 19 (42%) without CTCRD, *p* = 0.16]. There were no reports of symptomatic left ventricular systolic dysfunction (LVSD) according to CTCAE in subjects treated on AAML1421.

Additionally, significant declines were seen in CS following CPX-351 [baseline, −25.1% (IQR: −30.1, −21.9%); end of CPX-351 cycle −22.8% (IQR: −25.8, −18.9%); ΔCS 2.8% (IQR: 0.5, 5.3%), *p* = 0.03]. There was a numerical worsening LVFS [ΔLVFS −1.1% (IQR: −5.9, 1.9), *p* = 0.29] and global longitudinal strain [ΔGLS 0.9% (IQR: −1.8, 3%), *p* = 0.30] following CPX-351, although these changes were not statistically significant.

### 3.2 Changes in cardiac biomarkers of injury and stress

**Figure 2:**
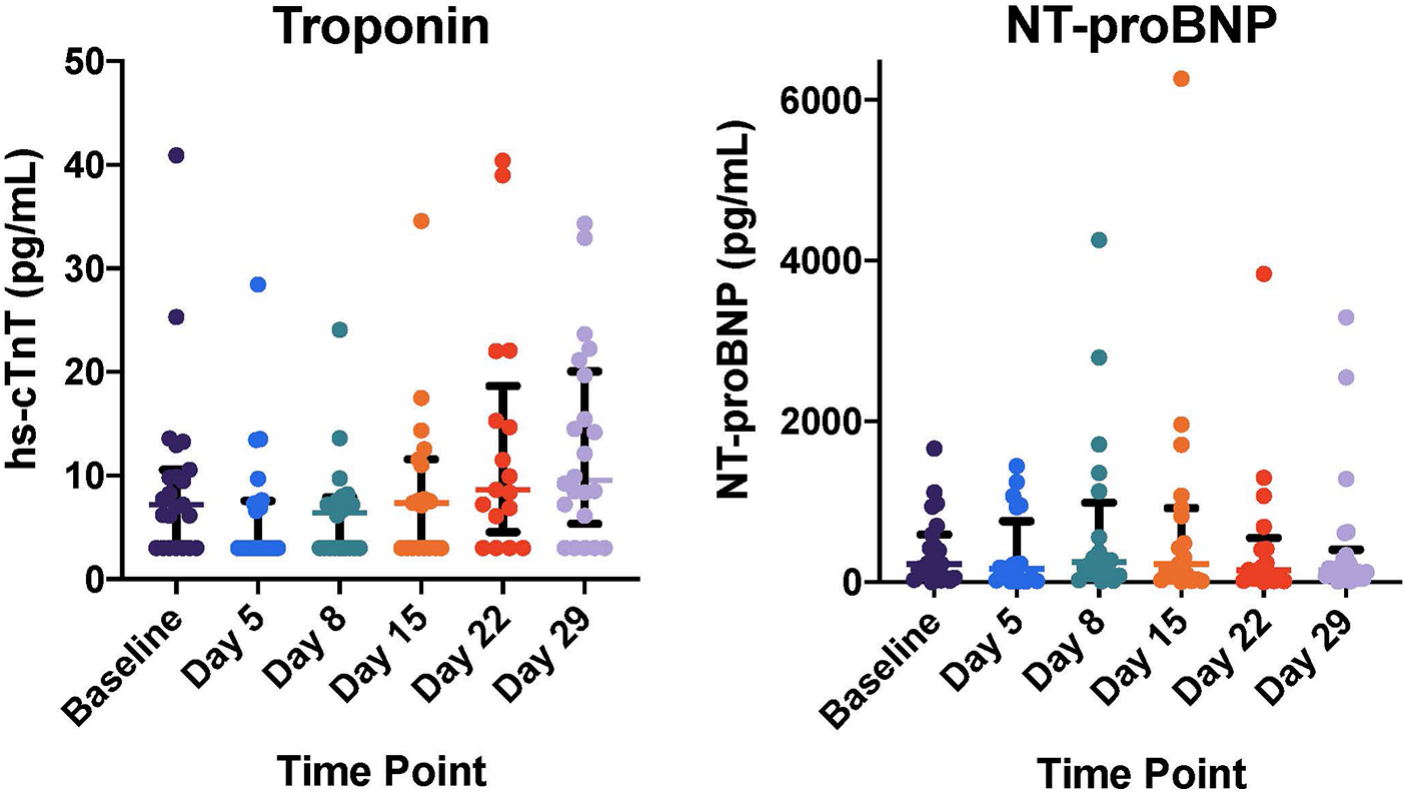
Cardiac biomarker levels following CPX-351 including high sensitivity cardiac troponin (hs-cTnT) and N-terminal pro-B-type natriuretic peptide (NT-proBNP). Individual patient biomarker measurements plotted with overlying median and interquartile range for each time point. cTnT increased over time post-CPX-351 and was modestly increased at cycle day 29 compared to baseline [Baseline 7.2 (IQR: 3.0, 10.6); Day 29, 9.6 (IQR: 6.1, 19.7); ΔcTnT 1.80 (IQR: 0, 6.1), *p* = 0.03]. NT-proBNP did not change significantly following CPX-351.

Baseline elevations in cardiac biomarkers were common prior to initiation of CPX-351 with abnormal hs-cTnT and NT-proBNP in 12 (48%) and 14 (56%) of 25 subjects, respectively. Hs-cTnT increased over time post-CPX-351 and was modestly increased at cycle 1 day 29 compared to baseline [Baseline 7.2 (IQR: 3.0, 10.6); Day 29, 9.6 (IQR: 6.1, 19.7); ΔcTnT 1.80 (IQR: 0, 6.1), *p* = 0.03, Figure 2A]. NT-proBNP remained elevated without significant change following CPX-351 (Figure 2B). No statistically significant associations were observed between baseline or post-CPX-351 biomarkers and end of CPX-351 cycle LVEF.

## 4 Discussion

In effort to address the critical need for more effective AML salvage regimens with lower cardiotoxicity risk, the Children's Oncology Group carried out this phase I/II study of CPX-351 and demonstrated one of the highest response rates reported to date in children and adolescents with relapsed AML with a CR + CRp rate of 68.3% (90% CI: 52.9%–78.0%) (7). Central echo quantitation in this heavily anthracycline pre-treated population demonstrated a high prevalence of subclinical cardiac dysfunction at study entry with LVEF of less than 50% in 8 of 25 pts (32%) despite trial inclusion criteria requiring adequate cardiac function based on LVEF measured by the treating institution. Similarly, nearly half of these subjects demonstrated elevations in hs-cTnT and/or NT-proBNP at trial enrollment. Receipt of CPX-351 containing reinduction resulted in significant short-term declines in LVEF and increases in hs-cTnT. Six of 25 (24%) analyzable subjects experienced cancer therapy related cardiac dysfunction (CTCRD) at the end of reinduction cycle 1 (defined as LVEF decline of ≥10%–<50%). While the limited sample size precluded identification of clinical predictors of cardiotoxicity, female sex and occurrence of grade 3+ infection during cycle 1 were more frequently observed in subjects with CTCRD compared to those without. These findings are consistent with other studies of childhood cancer survivors demonstrating a greater cardiotoxicity risk in female survivors of childhood cancer (11, 12), as well as higher risk for LVSD in subjects experiencing high grade infections following chemotherapy (13). Higher cardiotoxicity risk in females is not fully understood, however may relate in part to differences in body composition between females and males. Higher body fat content, more commonly seen in females, has been associated with reduced anthracycline clearance which may contribute to greater drug exposure and toxicity (14, 15). In our cohort, overweight or obese status was more common in female pediatric participants [5 of 11 (45.5%)] compared make participants [4 of 14 (28.6%), *p* = 0.434]. Both duration of anthracycline exposure (i.e., area under the curve) and peak anthracycline concentrations have been shown to influence cardiotoxicity, though their relevance to liposomal encapsulated anthracyclines with reduced cardiac tissue penetration is not fully understood (16, 17).

While the high prevalence of abnormal LVEF and cardiac biomarkers at study entry was surprising, particularly given the requirement for normal cardiac function for study enrollment, 68% of the study population enrolled within 1 year of first remission. Thus, these findings are likely to reflect residual cardiac effects from recent anthracycline, potentially exacerbated by acute stressors associated with active relapse (i.e., hyperleukocytosis, sepsis, etc.). Existing data from the two most recent COG studies in *de novo* AML demonstrate significant cardiac dysfunction in approximately 10% of children during the first year of follow-up after frontline therapy (1, 13). AML relapse and the receipt of salvage therapy results in even higher rates of cardiotoxicity with early (during or within one year of salvage therapy) and late cardiotoxicity (beyond the first year post-treatment) seen in up to 20% and 37% of survivors with relapsed AML, respectively (18). Unfortunately, data comparing the cardiotoxicity across salvage regimens for pediatric AML are not available. While cardiotoxicity risk must be carefully considered when pursuing relapse therapy, anti-leukemic efficacy and survival is essential. Pediatric studies have demonstrated superior early treatment response with use of anthracycline based AML salvage regimens compared to similarly intensive non-anthracycline based regimens (19). Thus, anthracyclines are commonly utilized during first salvage for children with relapsed AML.

Liposomal encapsulation of anthracyclines has shown to be an effective strategy to maintain the efficacy of anthracycline based regimens with less cardiotoxicity (20, 21). Early findings in adults with newly diagnosed high-risk or secondary AML indicate lower rates of cardiotoxicity with CPX-351 compared to standard anthracyclines (5, 6). Post hoc central quantitation of serial echocardiograms in 102 of 300 adult participants on this randomized trial of CPX-351 demonstrated lower rates of clinically significant reduction in LVEF (8.8% vs. 20%) or GLS (21% vs. 44%) with CPX-351 vs. daunorubicin/cytarabine at a median follow up of approximately 6 months (5). AAML1421 is the first study to describe the cardiotoxic effects of CPX-351 in children. While evidence of cardiotoxicity was common, this small, single arm study cannot inform to what degree the troponin elevations and LVEF reduction were directly related to CPX-351 vs. pre-existing cardiac dysfunction and/or high-grade infections/sepsis. Sepsis, which can result in acute cardiac decompensation even in the absence of anthracyclines, is known to increase cardiotoxicity risk in *de novo* pediatric AML cohorts (13, 22). Absent a comparator population, it is not clear how the post-CPX-351 trends in cardiac function and biomarkers observed in this study compare to non-liposomal anthracycline or non-anthracycline salvage regimens for pediatric AML. To understand the cardiac effects of CPX-351 compared to standard anthracyclines in children with AML, the most rigorous design is to study this in a randomized fashion in anthracycline naïve children. To this end, the COG is currently conducting a randomized study of CPX-351 vs. standard daunorubicin-containing induction given with dexrazoxane, which has emerged as an effective cardioprotective strategy in pediatric AML (1) (ClinicalTrials.gov; NCT04293562). Notably, dexrazoxane dosing strategies have not been studied with liposomal formulations of anthracycline given its short half-life compared to the prolonged drug exposure of liposomal encapsulated anthracycline, and thus, will not be given with CPX-351 (3). Prospective evaluation and comparison of cardioprotective strategies via detailed core lab quantitated echocardiography and cardiac biomarkers is an integral aim of this trial. In addition, pharmacokinetic (PK) profiling of CPX-351 is being performed and will enhance our understanding of the role of anthracycline PK parameters (i.e., AUC and peak concentration) in cardiotoxicity risk.

Discordance between core lab and clinical site quantitation of LV function at study entry is well-recognized in the field of cardiology (23). In our study, core lab quantitated LVEF was consistently lower than site quantitated LVEF with a median difference of 11% [IQR: 6.7–13.4%]. Discordance was similar between baseline and end of cycle 1 time points. Interestingly, only one patient met criteria for CTRCD based on site quantitated LVEF (as expected, this patient also met criteria for CTRCD based on core lab quantitation). Abundant literature exists supporting the superiority of core lab interpretations for reducing variability and enhancing the precision of echocardiographic measurements, thereby allowing for more sensitive detection of therapy-induced change over time (23–27). Further, these data indicate that real time core lab quantitation of baseline LVEF may be a necessary component of trial eligibility to ensure adequate cardiac function when studying potentially cardiotoxic agents in very high risk anthracycline-pre-treated children.

Notably, the early timing of post-CPX-351 cardiac assessments and the lack of availability of late echocardiographic images for analysis limit our ability to understand whether these short-term indicators of cardiotoxicity predict late cardiovascular morbidity. While many patients will experience LVEF recovery, particularly with institution of cardiac supportive care, LVSD during AML therapy have been associated with increased risk for persistent or recurrent LVSD in follow-up (13, 28). Hence, late cardiomyopathy remains a significant concern in this high risk population.

## 5 Conclusion & future directions

Children with relapsed AML have high rates of subclinical cardiac dysfunction prior to initiating salvage therapy. The administration of CPX-351 resulted in short term declines in LV function and elevations in hs-cTnT indicative of cardiotoxic effects. However, absent a comparator population, it is not known how these toxicities compare with alternative salvage regimens for pediatric AML. To better understand the cardioprotective effect of CPX-351, COG is currently conducting a phase 3 randomized trial of CPX-351 in children and adolescents with *de novo* AML. This trial aims to elucidate the optimal cardioprotective strategies in the upfront setting such that in the unfortunate event of relapse, children are better able to tolerate salvage therapy while maintaining long-term cardiovascular health.

## Data Availability

The original contributions presented in the study are included in the article/supplementary material, further inquiries can be directed to the corresponding author/s. The AAML1421 protocol and study material can be made available upon request.
