# Supplementary material for: Cardiotoxicity of CPX-351 in children and adolescents with relapsed AML: a Children's Oncology Group report
Source: Front Cardiovasc Med. 2024 Jun 14;11:1347547. doi: 10.3389/fcvm.2024.1347547 (PMC11211570; doi:10.3389/fcvm.2024.1347547)
Supplement: Supplementary file 1 [file Datasheet1.docx]

Supplementary Material

# Supplementary Figures

#
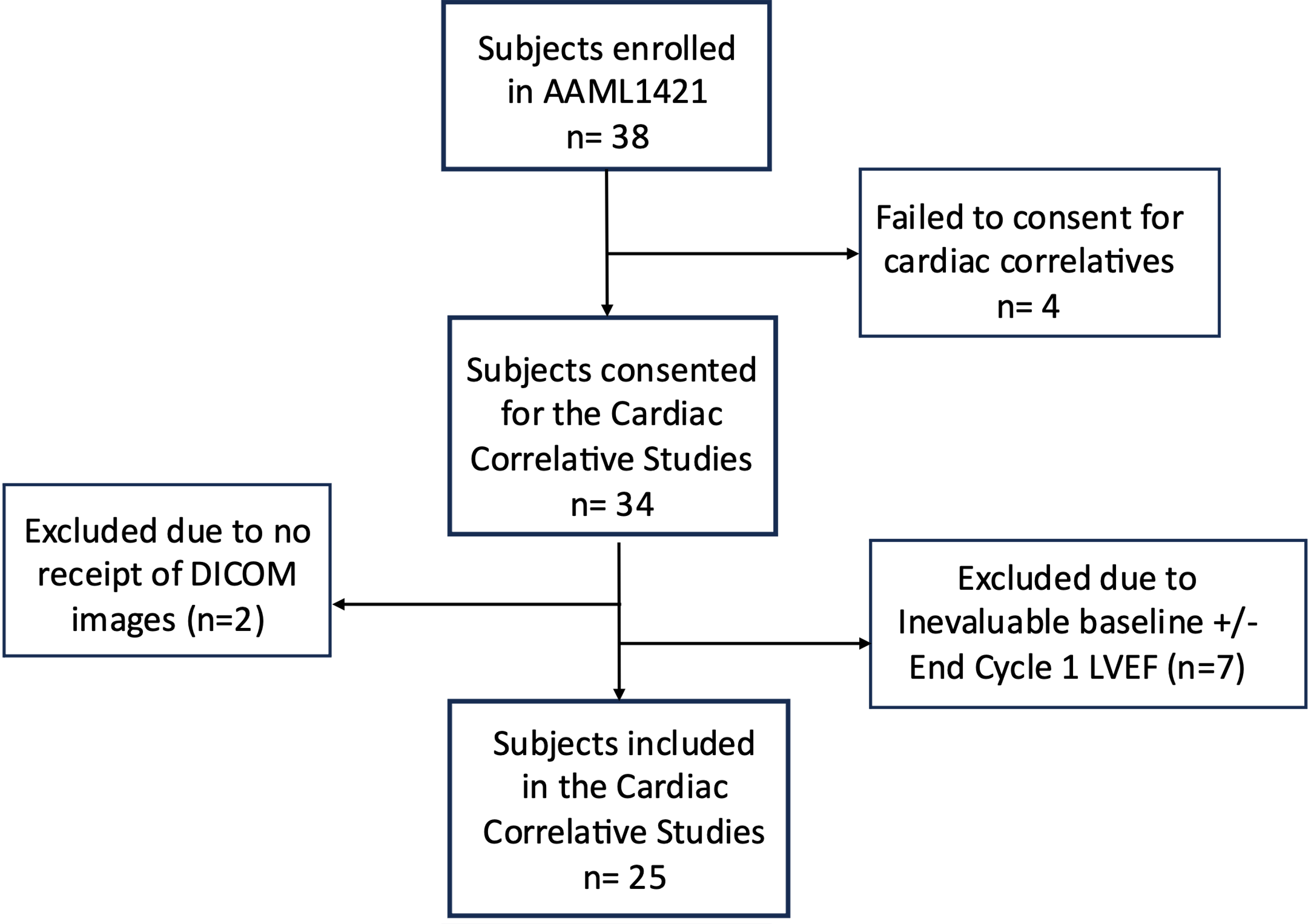


**Supplemental Figure 1. Consort Diagram for Subjects Enrolled vs. Included in the Cardiac Correlative Studies**
